# Inherited bone marrow failure syndromes in childhood: clinical presentation, diagnostic approach, and contemporary management

**DOI:** 10.3389/fped.2026.1909640

**Published:** 2026-10-27

**Authors:** Simona Ivančan, Janez Jazbec

**Affiliations:** 1 Department of Pediatrics, University Medical Centre Ljubljana, Ljubljana, Slovenia; 2 Faculty of Medicine, University of Ljubljana, Ljubljana, Slovenia

**Keywords:** clonal evolution, GATA2 deficiency, germline predisposition, hematopoietic stem cell transplantation, inherited bone marrow failure syndromes, next-generation sequencing, pediatric cytopenia, SAMD9/SAMD9L

## Abstract

**Background:**

Inherited bone marrow failure syndromes (IBMFS) comprise a heterogeneous group of rare genetic disorders defined by intrinsic defects of hematopoietic stem cells, resulting in chronic cytopenias, congenital anomalies, and a markedly increased risk of myelodysplastic syndrome (MDS), acute myeloid leukemia (AML), and solid malignancies. Despite their clinical significance, IBMFS remain substantially underdiagnosed due to phenotypic heterogeneity, incomplete penetrance, and overlap with acquired marrow failure.

**Methods:**

This narrative review synthesizes current evidence on the pathogenesis, clinical manifestations, differential diagnosis, clonal evolution, and syndrome-specific management of major IBMFS in pediatric patients, with emphasis on recently published data and areas of active clinical controversy.

**Results:**

Advances in next-generation sequencing (NGS) have expanded the recognized spectrum of IBMFS and demonstrated that germline predisposition underlies a significant proportion of cases initially classified as acquired marrow failure. Germline defects affecting DNA repair (Fanconi anemia, now comprising 23 complementation groups including the recently described FANCX), telomere maintenance (dyskeratosis congenita and telomere biology disorders), and ribosome biogenesis (Diamond–Blackfan anemia, Shwachman–Diamond syndrome) converge on shared pathogenetic mechanisms of hematopoietic stem cell exhaustion and clonal selection. GATA2 deficiency represents a transcription factor disorder with distinctive immunological and malignant features and is mechanistically related to SAMD9/SAMD9L-associated disorders through shared chromosome 7 instability. Congenital amegakaryocytic thrombocytopenia and severe congenital neutropenia represent additional single-lineage IBMFS that are frequently overlooked but carry their own malignant potential. Clonal evolution in IBMFS is biologically distinct from age-related clonal hematopoiesis and requires syndrome-specific surveillance and risk stratification. Hematopoietic stem cell transplantation (HSCT) remains the only curative option for hematologic disease but requires individualized conditioning and timing.

**Conclusion:**

Early recognition of IBMFS through systematic integration of clinical red flags and genetic diagnostics helps prevent diagnostic delays, guide appropriate management, inform timing of HSCT, and support lifelong multidisciplinary surveillance.

## Highlights

**What is Known:**

- Inherited bone marrow failure syndromes (IBMFS) are rare genetic disorders causing progressive cytopenias, congenital anomalies, and markedly elevated risk of MDS and AML.
- Distinguishing IBMFS from acquired aplastic anemia is clinically important, as misclassification can lead to ineffective or harmful treatment and suboptimal HSCT conditioning.

**What is New:**

- Next-generation sequencing has demonstrated that germline predisposition underlies up to 20%–35% of pediatric aplastic anemia and MDS cases, substantially expanding the recognized disease spectrum.
- Pre-emptive HSCT before overt MDS development, emerging gene therapies (notably for Fanconi anemia), and error-corrected deep sequencing for clonal surveillance represent key recent advances with direct clinical implications.
- A 23rd Fanconi anemia complementation group (FANCX/FAAP100) and an expanded panel of telomere biology disorder genes have recently been described, and SAMD9/SAMD9L-related disorders, congenital amegakaryocytic thrombocytopenia, and severe congenital neutropenia are increasingly recognized as clinically important IBMFS that overlap mechanistically with GATA2 deficiency and chromosome 7 instability.

## Introduction

Inherited bone marrow failure syndromes (IBMFS) represent a group of genetic disorders in which intrinsic defects of hematopoietic stem cells result in ineffective hematopoiesis, chronic cytopenias, and a substantially elevated lifetime risk of myeloid malignancies (1, 2). These disorders are frequently associated with congenital anomalies, immune dysfunction, and multisystem involvement, rendering clinical presentation highly variable and diagnostic recognition challenging.

Despite their clinical importance, IBMFS remain substantially underdiagnosed. Phenotypic heterogeneity, incomplete penetrance, and considerable overlap with acquired marrow failure—most notably aplastic anemia and pediatric MDS—often lead to delayed or incorrect diagnosis, with direct consequences for patient management. Misclassification as acquired aplastic anemia may result in the administration of immunosuppressive therapy, which is not only ineffective but potentially harmful in selected IBMFS such as Fanconi anemia. Failure to recognize an inherited etiology may also lead to inappropriate donor selection for HSCT, use of inadequately modified conditioning regimens, and suboptimal surveillance for malignant and extrahematologic complications (3, 4). A particularly consequential pitfall is the inadvertent use of an asymptomatic relative who unknowingly carries the same germline mutation as a hematopoietic stem cell donor; this can transfer the underlying IBMFS into the graft, resulting in early graft dysfunction, donor-derived clonal disease, or transplant of an equally susceptible stem cell pool, and underscores the value of germline testing of related donors before transplantation (3, 5).

Over the past decade, next-generation sequencing has transformed the diagnostic landscape, demonstrating that germline predisposition underlies a clinically significant proportion of cases initially considered acquired, and enabling identification of an expanding spectrum of predisposition syndromes (4, 5). Systematic sequencing cohorts suggest that up to 20%–35% of children with aplastic anemia or pediatric MDS harbor an underlying germline mutation (3, 6).

## Methods

This is a narrative review. We searched PubMed and Embase for English-language publications from January 2015 through June 2026, supplemented by manual screening of the reference lists of retrieved articles and of major reviews and guidelines; foundational studies published before 2015 were retained where they remain the primary or most relevant source for a given mechanism or finding. Search terms combined “inherited bone marrow failure syndrome(s)” and “bone marrow failure” with terms for individual disorders—Fanconi anemia, dyskeratosis congenita/telomere biology disorders, Diamond–Blackfan anemia, Shwachman–Diamond syndrome, GATA2 deficiency, SAMD9/SAMD9L-related disorders, congenital amegakaryocytic thrombocytopenia, and severe congenital neutropenia—combined with terms relating to pathogenesis, diagnosis, clonal evolution, and hematopoietic stem cell transplantation. Systematic reviews, comprehensive narrative reviews, original research studies, and major consensus or guideline statements were prioritized. Studies were selected for relevance to pediatric populations; literature describing predominantly adult-onset manifestations or outcomes was included only where necessary to inform the natural history, surveillance, or long-term consequences relevant to children and adolescents. Given the breadth and rarity of the disorders covered and the narrative format of this review, no formal systematic methodology (e.g., PRISMA) or quality-scoring system was applied; the goal was a clinically oriented, evidence-informed synthesis rather than an exhaustive systematic appraisal.

## Epidemiology and clinical significance

The combined incidence of the classic IBMFS is estimated at approximately 1 in 15,000 (64.5 cases per million births in the largest population-based cohort to date) live births, although this figure likely underestimates true prevalence (1, 7). Many affected individuals present with mild, isolated, or slowly progressive cytopenias and are diagnosed only in later childhood, adolescence, or even adulthood. In syndromes characterized by incomplete penetrance or late-onset hematologic manifestations—most notably telomere biology disorders and GATA2 deficiency—clinical recognition may be further delayed by the absence of overt congenital features at presentation.

IBMFS constitute a major subgroup of inherited cancer predisposition syndromes, with substantially elevated risk of MDS, AML, and selected solid tumors (2, 8). The risk of malignant transformation is not uniformly correlated with the severity of hematologic disease: clonal evolution may occur even in patients with mild or compensated cytopenias. Successful HSCT corrects the hematopoietic compartment and eliminates the risk of recipient-derived MDS/AML arising from the patient's own marrow, but it does not remove the underlying germline defect from non-hematopoietic tissues; consequently, the risk of solid malignancy—rather than hematologic malignancy—persists or may even increase after HSCT, underscoring the need for long-term surveillance (2, 9). The magnitude of malignant risk varies considerably across syndromes, being particularly high in Fanconi anemia (cumulative incidence of bone marrow failure, MDS/AML, or solid malignancy approaching 90% by age 50, although the risk of MDS/AML specifically is estimated at approximately 30% by age 40, with solid tumors accounting for a substantial share of later lifetime risk (10, 11)), GATA2 deficiency (approximately 70%), and Shwachman–Diamond syndrome (approximately 30%–35%). Progressive marrow failure necessitates recurrent transfusions, growth factor therapy, and eventually HSCT; multisystem involvement imposes additional morbidity requiring coordinated multidisciplinary care.

## Critical challenges in diagnosis and management

### Distinguishing IBMFS from acquired marrow failure

The clinical differentiation of IBMFS from acquired aplastic anemia or pediatric MDS remains one of the most consequential diagnostic challenges in pediatric hematology. This challenge is particularly acute in patients who lack overt congenital anomalies or a positive family history, which is the case in a clinically significant proportion of affected individuals. In telomere biology disorders and GATA2 deficiency, syndromic features may be absent or develop only later, and the hematologic presentation may be indistinguishable from acquired marrow failure (5, 12, 13). Figure 1 presents a diagnostic algorithm for the systematic evaluation of a child presenting with cytopenia(s), integrating these clinical and laboratory clues.

**Figure 1:**
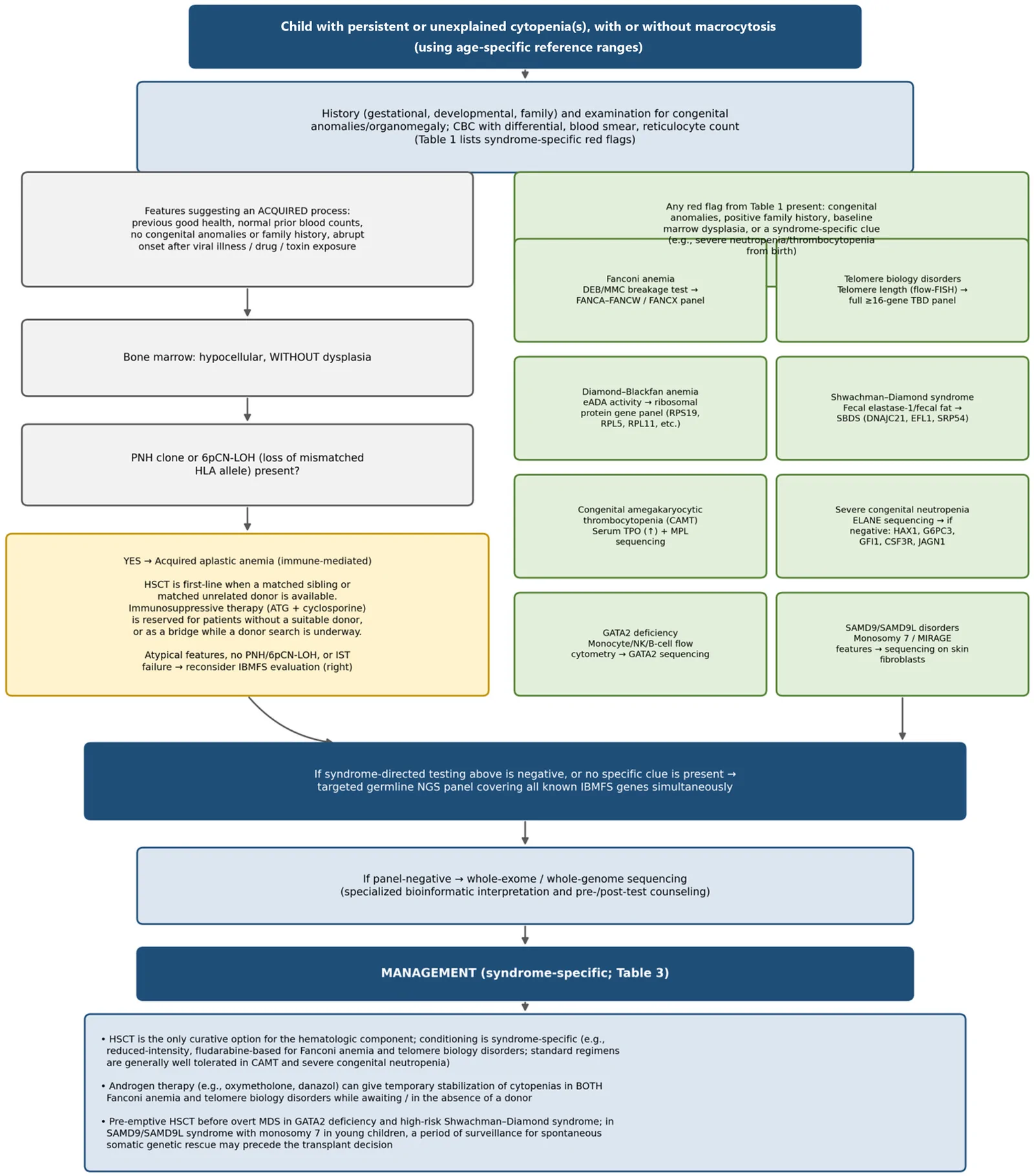
Diagnostic algorithm for a child presenting with cytopenia(s). Left branch: clinical/laboratory pattern suggesting acquired aplastic anemia, including the role of a PNH clone or 6pCN-LOH. Right branch: syndrome-directed first-tier testing for the eight major IBMFS discussed in the text, escalating to a germline NGS panel and, if negative, whole-exome/whole-genome sequencing. The management box summarizes HSCT and androgen therapy considerations detailed in Table 3.

The clinical impact of misclassification is substantial. Immunosuppressive therapy with antithymocyte globulin (ATG) and cyclosporine—standard first-line treatment for acquired aplastic anemia—is not only ineffective in IBMFS but may be harmful. This risk is particularly acute in telomere biology disorders, where many patients harbor an occult T-cell immunodeficiency independent of overt marrow failure, so that ATG-based immunosuppression can precipitate life-threatening infectious complications (14). In Fanconi anemia, exposure to DNA-damaging agents in diagnostic or therapeutic settings carries serious risks. Inappropriate conditioning regimens in unrecognized IBMFS patients proceeding to HSCT are associated with significantly increased transplant-related toxicity and mortality (15, 16).

### Timing of hematopoietic stem cell transplantation

The optimal timing of HSCT in IBMFS remains an area of active controversy. Early transplantation reduces the risk of clonal evolution and irreversible organ damage; however, it must be weighed against procedure-related morbidity and mortality, which are significantly elevated in certain syndromes (15–17). In Fanconi anemia, hypersensitivity to alkylating agents and radiation generally necessitates modified, reduced-intensity conditioning protocols. In telomere biology disorders, pre-existing pulmonary fibrosis or hepatic dysfunction substantially increases transplant-related toxicity and may contraindicate standard-intensity regimens. Emerging data support pre-emptive HSCT prior to overt MDS in GATA2 deficiency and high-risk SDS, though prospective evidence remains limited.

Clonal hematopoiesis occurs in IBMFS through a biologically distinct mechanism from age-related clonal hematopoiesis of indeterminate potential (CHIP). In IBMFS, chronic cellular stress and reduced hematopoietic reserve create strong selective pressure for acquisition of somatic mutations that confer clonal survival advantage. Not all emergent clones carry equivalent malignant risk, and distinguishing high-risk clonal evolution from indolent or adaptive clonal hematopoiesis constitutes a major clinical challenge (8, 18). Somatic mutations in TP53, RUNX1, and ASXL1 indicate high malignant risk; in SDS, iso(7q) may be relatively indolent while monosomy 7 confers high risk. These general principles take disease-specific forms. In Fanconi anemia, gain of chromosome 1q is the most common and earliest route to clonal dominance, driving MDM4 trisomy that attenuates the chronically elevated p53 response otherwise limiting FA hematopoietic stem cells, while cryptic RUNX1 mutations constitute a second, later-arising leukemogenic pathway found in up to one-fifth of patients (19). In GATA2 deficiency, monosomy 7 and trisomy 8 are the dominant cytogenetic routes to MDS, occurring in roughly 70% and 24% of mutation carriers respectively, with acquisition of ASXL1 or STAG2 mutations marking further clonal progression (20, 21). In SAMD9/SAMD9L syndromes, clonal hematopoiesis arises through somatic genetic rescue of the growth-suppressive germline variant, which may be adaptive (revertant uniparental disomy of chromosome 7q, or a second-site SAMD9/SAMD9L mutation restoring normal function) or maladaptive (monosomy 7, often with acquisition of additional cancer-associated mutations); the two trajectories carry markedly different prognostic implications despite a shared starting karyotype (22). In severe congenital neutropenia, a two-step model predominates: truncating CSF3R mutations arise first, conferring a proliferative advantage under chronic G-CSF exposure, and are followed—often years later—by RUNX1 mutations that mark imminent transformation to MDS/AML (23).

## Pathogenesis

Despite substantial genetic heterogeneity, IBMFS share a common pathogenetic outcome: progressive exhaustion of hematopoietic stem cell self-renewal and differentiation capacity, leading to ineffective hematopoiesis, cytopenias, and selective pressure for clonal evolution (5). Three principal molecular pathways are affected across the major syndromes.

### Defects in DNA repair

Fanconi anemia is the prototypical disorder of DNA interstrand crosslink repair, resulting from biallelic mutations in genes constituting the FA/BRCA pathway (FANCA through FANCW, together with the recently identified FANCX/FAAP100, bringing the total to 23 known complementation groups) (10, 24), although FANCB is inherited in an X-linked recessive manner and FANCR/RAD51 is autosomal dominant, unlike the biallelic recessive pattern of the other complementation groups. Failure to resolve interstrand crosslinks—caused by endogenous aldehydes and exogenous genotoxic exposure—leads to chromosomal breakage, cell cycle arrest, and apoptosis. Hematopoietic stem cells are disproportionately vulnerable due to high proliferative demands and sensitivity to aldehyde-induced DNA damage. Genomic instability progressively depletes the functional stem cell pool and fosters acquisition of secondary somatic mutations, driving clonal evolution toward MDS and AML.

### Defects in telomere maintenance

Telomere biology disorders arise from mutations in genes encoding components of the telomerase holoenzyme (DKC1, TERC, TERT, NOP10, NHP2, TCAB1/WRAP53, NAF1) or telomere-protective shelterin and CST-complex proteins (RTEL1, TINF2, CTC1, STN1, ACD, POT1, PARN, RPA1, ZCCHC8, DCLRE1B) (25, 26). At least 16 genes are now established causes of dyskeratosis congenita/telomere biology disorders, with additional candidate loci (NPM1, MDM4, POLA1) reported in dyskeratosis congenita-like phenotypes (25). These mutations accelerate telomere attrition, leading to premature cellular senescence and p53-dependent apoptosis. Hematopoietic stem cells, which depend on sustained proliferative capacity for lifelong blood cell production, are particularly susceptible to critically short telomeres. The resulting progressive bone marrow failure may be compounded by multiorgan involvement—including pulmonary fibrosis, portal hypertension, and liver cirrhosis—reflecting the fundamental importance of telomere integrity in high-turnover tissues; however, these extrahematologic manifestations are predominantly features of adult-onset disease and are uncommon at the time of presentation in children, in whom immunodeficiency and bone marrow failure are instead the most common presenting manifestations (27).

### Defects in ribosome biogenesis (ribosomopathies)

Diamond–Blackfan anemia and Shwachman–Diamond syndrome are the two major ribosomopathies causing IBMFS (28, 29). In Diamond–Blackfan anemia, haploinsufficiency of ribosomal protein genes (most commonly RPS19, RPL5, and RPL11) impairs ribosome assembly, activates p53-dependent stress pathways, and leads to selective apoptosis of erythroid progenitors. In Shwachman–Diamond syndrome, mutations in SBDS (and less commonly DNAJC21 or EFL1) disrupt ribosome maturation, causing broader hematopoietic progenitor dysfunction alongside exocrine pancreatic insufficiency.

### GATA2 deficiency: A transcription factor disorder

GATA2 deficiency constitutes a distinct pathogenetic category, arising from heterozygous loss-of-function mutations in the GATA2 transcription factor gene (30). GATA2 regulates differentiation of hematopoietic stem and progenitor cells and is essential for maintenance of monocyte, dendritic cell, B cell, and NK cell lineages. Haploinsufficiency results in a stereotyped pattern of selective cytopenias—most notably monocytopenia and lymphopenia—that underlie both the immunodeficiency phenotype and the very high lifetime risk of myeloid transformation.

### SAMD9/SAMD9L-related disorders: A mechanistic link to chromosome 7 instability

SAMD9 and SAMD9L are adjacent, interferon-regulated genes on chromosome 7q21.2 that normally restrain cell growth. Germline gain-of-function mutations produce excessive antiproliferative signaling in hematopoietic progenitors, causing marrow failure that is frequently “rescued” by somatic clones that have lost the mutant allele—most often through loss of the entire chromosome 7 (monosomy 7), uniparental disomy 7q, or a second, inactivating somatic mutation in cis (31). This process of somatic genetic rescue, also termed adaptation by aneuploidy, explains the historically puzzling phenomenon of transient monosomy 7 in young children and links germline SAMD9/SAMD9L mutations mechanistically to a substantial proportion of pediatric MDS with monosomy 7, alongside germline GATA2 mutations (31). The clinical spectrum spans MIRAGE syndrome (myelodysplasia, infection, restriction of growth, adrenal hypoplasia, genital phenotypes, enteropathy), ataxia-pancytopenia syndrome, and isolated familial or sporadic pediatric MDS/aplastic anemia with monosomy 7.

## Clinical presentation

The clinical presentation of IBMFS is remarkably heterogeneous and represents a major contributor to diagnostic delay. Manifestations range from mild, isolated cytopenias incidentally detected on routine blood counts to severe pancytopenia presenting acutely in infancy. Disease course may be insidious and prolonged, with periods of relative stability interrupted by progressive hematopoietic deterioration or abrupt development of clonal complications (1, 13).

### Hematologic manifestations

The most common initial feature is cytopenia of one or more hematopoietic lineages. Macrocytosis is a helpful early clue in several IBMFS—reflecting ineffective, stress-driven erythropoiesis—and in disorders such as Fanconi anemia and Diamond–Blackfan anemia it frequently precedes the onset of overt cytopenia. However, macrocytosis should be interpreted against age-specific pediatric reference ranges for mean corpuscular volume rather than a fixed adult threshold such as MCV >100 fL, since normal ranges in infants and young children are substantially lower and a fixed cutoff can miss clinically relevant macrocytosis in this age group. Macrocytosis is also neither a universal nor a sufficiently specific finding across IBMFS: several syndromes, including congenital amegakaryocytic thrombocytopenia, severe congenital neutropenia, and early Shwachman–Diamond syndrome, characteristically present instead with isolated thrombocytopenia or neutropenia rather than macrocytic change, and GATA2 deficiency typically presents with selective lymphoid and monocytic cytopenias without a preceding macrocytic phase. When present and otherwise unexplained for age, macrocytosis should prompt consideration of an underlying IBMFS, but its absence does not exclude one, and lineage-specific findings should be weighed on their own merits. In Diamond–Blackfan anemia, selective impairment of erythropoiesis predominates, producing severe macrocytic anemia with profound reticulocytopenia within the first year of life, while granulopoiesis and thrombopoiesis are initially preserved. Shwachman–Diamond syndrome characteristically presents first with chronic neutropenia, which may be mild at onset but progressively involves additional lineages. GATA2 deficiency, by contrast, typically presents later—in adolescence or adulthood—with a selective pattern of monocytopenia and B- and NK-lymphopenia rather than neutropenia, and neutrophil counts are often preserved until late in the disease course. Two further single-lineage IBMFS present characteristically in early infancy: congenital amegakaryocytic thrombocytopenia manifests as isolated, severe thrombocytopenia at or shortly after birth with absent or markedly reduced megakaryocytes, while severe congenital neutropenia presents with profound neutropenia (absolute neutrophil count typically <0.2 × 10^9^/L) and recurrent bacterial infections from the first months of life; both may evolve toward multilineage marrow failure if unrecognized.

### Congenital and extrahematologic manifestations

Congenital anomalies constitute important diagnostic clues but are neither universal nor pathognomonic within individual syndromes. Radial ray defects, renal malformations, cardiac anomalies, and short stature are characteristic of Fanconi anemia, while craniofacial abnormalities and thumb defects are associated with Diamond–Blackfan anemia. In dyskeratosis congenita, the classic clinical triad of reticular skin hyperpigmentation, nail dystrophy, and oral leukoplakia may be absent or develop only gradually; in severe forms such as Hoyeraal–Hreidarsson syndrome and Revesz syndrome, the presentation in infancy is dominated by neurological and ocular features.

### Immune dysfunction and infectious complications

Immune deficiency is an integral feature of several IBMFS, particularly GATA2 deficiency, which is characterized by severe depletion of monocytes, NK cells, and B lymphocytes, predisposing to nontuberculous mycobacteria (NTM), invasive fungal disease, and disseminated HPV-associated disease. Infection episodes may precede or occur independently of overt cytopenia, and recurrent NTM infection in a child or young adult should prompt consideration of GATA2 evaluation.

## When to suspect ibmfs: clinical red flags

Table 1 summarizes key clinical and laboratory features that should prompt systematic evaluation for an inherited etiology in a child presenting with cytopenia.

**Table 1:** Clinical and laboratory red flags for inherited bone marrow failure syndromes.

Clinical or laboratory red flag	Syndrome(s) to consider	Key syndrome-specific confirmatory test
Unexplained macrocytosis (above the age-specific reference range), with or without cytopenia	Consider an IBMFS (an early clue in some, but not all, syndromes)	Fetal hemoglobin, eADA, telomere length (flow-FISH), DEB/MMC breakage test, germline NGS panel
Radial ray defects, renal/cardiac anomalies, short stature, café-au-lait macules	Fanconi anemia	DEB/MMC chromosomal breakage test, then FA gene panel (*FANCA*–*FANCW*, *FANCX*)
Nail dystrophy, oral leukoplakia, reticular skin pigmentation (may be partial or absent); unexplained cytopenia with very short lymphocyte telomeres	Dyskeratosis congenita/telomere biology disorders (pulmonary fibrosis and liver disease are adult-onset features and uncommon at pediatric presentation)	Lymphocyte telomere length by flow-FISH (<1st percentile), followed by the full TBD gene panel (≥16 genes), not a limited subset
Severe macrocytic anemia with reticulocytopenia in the first year of life ± craniofacial/thumb anomalies	Diamond–Blackfan anemia	Erythrocyte adenosine deaminase (eADA) activity; ribosomal protein gene panel (*RPS19*, *RPL5*, *RPL11*, and others)
Chronic neutropenia with steatorrhea/failure to thrive and metaphyseal skeletal dysplasia	Shwachman–Diamond syndrome	Fecal elastase-1/72 h fecal fat; *SBDS* (or *DNAJC21*, *EFL1*, *SRP54*) sequencing; skeletal imaging
Isolated severe thrombocytopenia at birth or in the first weeks of life, with absent/reduced megakaryocytes	Congenital amegakaryocytic thrombocytopenia (CAMT)	*MPL* sequencing; elevated serum thrombopoietin level
Severe neutropenia (ANC <0.2 × 10^9^/L) with recurrent bacterial infections from early infancy; promyelocyte maturation arrest	Severe congenital neutropenia (SCN)	*ELANE* sequencing (∼50%–60% of cases); if negative, *HAX1*, *G6PC3*, *GFI1*, *CSF3R*, *JAGN1*
Monocytopenia with B- and NK-lymphopenia; recurrent NTM, invasive fungal or HPV-associated disease; lymphedema	*GATA2* deficiency	Peripheral blood monocyte/NK/B-cell flow cytometry; *GATA2* sequencing
Transient or persistent monosomy 7/del(7q) in a young child; MIRAGE-spectrum features (growth restriction, adrenal hypoplasia, enteropathy) or ataxia with pancytopenia	*SAMD9*/*SAMD9L*-related disorders	*SAMD9*/*SAMD9L* sequencing on non-hematopoietic tissue (e.g., skin fibroblasts), as somatic reversion in blood may mask the germline variant
PNH clone or acquired 6pCN-LOH on cytogenetic/SNP-array analysis	Favors acquired aplastic anemia (immune-mediated), not an IBMFS	Flow cytometry for GPI-anchored proteins (PNH); SNP-array/HLA genotyping for 6pCN-LOH

For each red flag, the syndrome(s) most strongly suggested and the confirmatory test of choice are indicated. A PNH clone or acquired 6p copy-number-neutral loss of heterozygosity (6pCN-LOH) favors an acquired, immune-mediated process over an inherited syndrome.

## Differential diagnosis of cytopenias in childhood

Cytopenia in childhood encompasses a broad diagnostic spectrum, and its systematic evaluation requires integration of patient age, clinical context, disease course, and laboratory findings. A primary distinction to establish in a child with persistent or unexplained cytopenia is between acquired and inherited causes, as this determination substantially influences therapeutic management.

### Acquired bone marrow failure

Acquired aplastic anemia represents the most frequent cause of persistent multilineage cytopenia in children, typically presenting abruptly after viral illness, drug exposure, or environmental toxin contact in a child who was previously entirely well, with normal complete blood counts on prior testing and without congenital anomalies or family history. Bone marrow examination characteristically reveals severe hypocellularity without dysplasia—the absence of dysplastic features is an important distinguishing point, since dysplasia is frequently present even at baseline in IBMFS; however, this baseline dysplasia in IBMFS is typically mild and should be distinguished from the frank, multilineage dysplasia of overt MDS, as mild features alone are usually insufficient to establish an MDS diagnosis in these patients. Response to immunosuppressive therapy with ATG and cyclosporine supports an acquired immune-mediated mechanism and effectively excludes most IBMFS. Detection of a paroxysmal nocturnal hemoglobinuria (PNH) clone or acquired 6p copy-number-neutral loss of heterozygosity (6pCN-LOH, leading to loss of the mismatched HLA allele) provides strong evidence for an immune-mediated, acquired process rather than a germline IBMFS, as these clonal escape mechanisms reflect selection under an autoimmune marrow attack (32–35). A substantial proportion of pediatric MDS cases (estimated 30%–40%) arise on a germline predisposition background; cytogenetic abnormalities or a suggestive personal/family history should prompt targeted germline evaluation.

### Clonal hematologic disorders

Distinguishing IBMFS-associated clonal evolution from *de novo* pediatric MDS arising without a recognized germline predisposition syndrome—rather than restating the general biology of clonal evolution already discussed above—is the central practical task in this differential, since the implications for family counseling, related-donor selection, and long-term surveillance differ substantially between the two. Features favoring an underlying IBMFS over *de novo* disease include a history of chronic, often subtle cytopenia, macrocytosis, or dysplasia predating the MDS diagnosis by months to years; coexisting extrahematologic features such as short stature, mucocutaneous, skeletal, or exocrine pancreatic abnormalities; a family history of cytopenia, early-onset MDS/AML, or unexplained organ dysfunction; and a marrow that was already chronically hypocellular before the clonal population emerged. *De novo* pediatric MDS, in contrast, more typically arises in a previously well child without antecedent cytopenia, congenital anomalies, or family history, with clonal lesions representing a genuine first event rather than a step in a pre-existing process of marrow exhaustion. Cytogenetic findings alone cannot reliably separate the two entities, however: monosomy 7 and del(7q), the most frequent clonal abnormalities in childhood MDS, occur with comparable frequency in *de novo* and IBMFS-associated disease (9, 11). Because clinical and family history are frequently unrevealing even in genuine germline disease, germline testing should be strongly considered in children presenting with MDS regardless of apparent phenotype, rather than reserved only for those with syndromic features, and is best performed on a non-hematopoietic tissue such as cultured skin fibroblasts, since somatic reversion or clonal dominance in blood and marrow can mask an underlying germline variant (9, 33).

## Diagnostic approach

### Initial evaluation

The diagnostic workup of a child with suspected IBMFS begins with a thorough clinical history (gestational, developmental, and family history), meticulous physical examination for congenital anomalies and organomegaly, and a complete blood count with differential and reticulocyte count. Peripheral blood smear morphology may reveal macrocytosis, dysplastic features, or abnormal cell morphology suggestive of specific syndromes. Red cell adenosine deaminase (eADA) activity is a diagnostically useful biomarker in Diamond–Blackfan anemia, and telomere length measurement by flow-FISH in lymphocyte subsets is a sensitive screening test for telomere biology disorders; fetal hemoglobin is frequently elevated across several IBMFS but is a nonspecific marker of stress erythropoiesis and does not itself point to a specific diagnosis. In children with neutropenia accompanied by failure to thrive, steatorrhea, or skeletal abnormalities, assessment of exocrine pancreatic function—fecal elastase-1 and quantitative 72 h fecal fat (or serum trypsinogen/isoamylase)—should be performed to evaluate for Shwachman–Diamond syndrome.

### Bone marrow assessment

Bone marrow aspirate and trephine biopsy with cytogenetics and fluorescence *in situ* hybridization (FISH) for chromosomes 5, 7, and 8 are generally indicated in children with unexplained persistent cytopenia (36). In IBMFS, baseline marrow cellularity is typically normal to reduced, and dysplastic features may be present even in the absence of overt MDS; marrow cellularity is variable over the disease course and may decline further with advancing disease. Marrow hypercellularity is not a typical baseline finding in IBMFS and, when present, should raise concern for clonal evolution toward MDS rather than be regarded as reassuring. Clonal cytogenetic abnormalities—particularly monosomy 7, del(7q), and complex karyotypes—warrant prompt specialist review and genetic evaluation. Where available, targeted NGS of marrow for somatic mutations associated with clonal progression (e.g., TP53, RUNX1, ASXL1, SAMD9/SAMD9L) provides valuable additional information for risk stratification and should be incorporated into the diagnostic workup whenever feasible.

### Genetic diagnostics

Chromosomal breakage testing with diepoxybutane (DEB) or mitomycin C (MMC) remains the gold standard for the diagnosis of Fanconi anemia due to its high sensitivity and clinical specificity, and is generally recommended in children with unexplained marrow failure and/or suggestive congenital anomalies, ideally before or alongside broader sequencing panels (10, 11, 36). Targeted germline NGS panels—covering all known IBMFS genes simultaneously—are recommended as first-line genetic evaluation in clinical settings with access to such testing. Whole-exome or whole-genome sequencing may identify novel or atypical variants in gene-panel-negative patients but requires specialized bioinformatic interpretation and appropriate pre- and post-test counseling. Lymphocyte telomere length below the first percentile by flow-FISH is highly sensitive for telomere biology disorders and should prompt sequencing of the full telomere biology disorder gene panel (at least the 16 established genes) rather than a limited subset, since reliance on a small number of genes will miss a substantial fraction of cases.

Despite their central role, NGS-based approaches carry important limitations that should inform their clinical use and interpretation. Short-read gene-panel and exome sequencing have reduced sensitivity for structural variants—large deletions, duplications, and complex rearrangements—that account for a meaningful fraction of pathogenic variants in several IBMFS genes (for example, ribosomal protein gene deletions in DBA); dedicated copy-number analysis such as multiplex ligation-dependent probe amplification or exome-based copy-number calling should be pursued when a strong clinical phenotype persists despite a negative sequencing panel, since copy-number variants account for an estimated 10%–15% of pathogenic variants across this gene set (37). Deep intronic, regulatory, and other non-coding variants are systematically missed by exome-focused panels and may require genome sequencing or RNA-based splicing assays to resolve a panel-negative case with a compelling phenotype. Panel gene content is also a moving target: panels not regularly updated will miss recently described genes such as FANCX/FAAP100 or newer telomere biology disorder genes, underscoring the value of periodic reanalysis of unsolved cases as the causal gene list continues to expand.

A further, clinically consequential challenge is the interpretation of variants of uncertain significance (VUS), which are identified in a substantial proportion of patients undergoing broad germline testing and should not, on their own, be used to confirm or exclude an IBMFS diagnosis, guide HSCT donor selection, or alter conditioning intensity. Formal classification using the American College of Medical Genetics and Genomics/Association for Molecular Pathology framework, family segregation analysis, and functional assays—for example, DEB/MMC chromosomal breakage testing in a patient with a FANC-gene VUS, or telomere length measurement in a relative carrying a telomere-gene VUS—are important tools for resolving clinical significance before major management decisions are made (37). Variant classifications are revised over time as population and functional databases expand, so unsolved or VUS-only cases benefit from periodic reanalysis and re-contact with the family rather than a single, static report.

## Overview of major inherited bone marrow failure syndromes

Table 2 summarizes the comparative genetic, clinical, and malignant-risk features of the major IBMFS discussed in this section. Fanconi anemia (FA) is the most common IBMFS and serves as the prototype of DNA repair deficiency disorders. It results from biallelic (or hemizygous in X-linked FANCB) mutations in genes of the FA/BRCA pathway, which is responsible for recognition and repair of DNA interstrand crosslinks (10, 11). Twenty-three complementation groups have now been identified, including the recently described FANCX (FAAP100) (24), of which FANCA, FANCC, and FANCG account for the majority of cases in European populations.

**Table 2:** Comparative features of major inherited bone marrow failure syndromes.

Syndrome	Key gene(s)/inheritance	Hallmark clinical features	Malignancy risk
Fanconi anemia	*FANCA*–*FANCW*, *FANCX* (23 genes); Usually autosomal recessive; *FANCB* X-linked; *FANCR* (RAD51) autosomal dominant	Progressive pancytopenia (age 5–10y), macrocytosis, elevated fetal hemoglobin; congenital anomalies (radial ray, renal, short stature, café-au-lait macules) in ∼75%; DEB/MMC hypersensitivity	MDS/AML >30% by age 40; solid tumors (HNSCC, anogenital SCC) 30%–50%
Telomere biology disorders/dyskeratosis congenita	≥16 genes (*DKC1*, *TERC*, *TERT*, *TINF2*, *RTEL1*, *ACD*, *POT1*, *PARN*, and others); XLR, AD, or AR	Variable BMF (isolated cytopenia to severe aplastic anemia); mucocutaneous triad in <50%; very short lymphocyte telomeres (<1st percentile); PF/liver disease are adult-onset and uncommon in children	Elevated MDS/AML and HNSCC risk; magnitude varies by gene/genotype
Diamond–Blackfan anemia	*RPS19* (∼25%), *RPL5*, *RPL11*, >20 genes; mostly AD	Severe macrocytic anemia with reticulocytopenia <1y; elevated eADA; craniofacial, thumb (triphalangeal), cardiac, urogenital anomalies in ∼50%	Increased MDS/AML and osteosarcoma risk; annual surveillance from adolescence
Shwachman–Diamond syndrome	*SBDS* (∼90%), *DNAJC21*, *EFL1*, *SRP54*; AR	Chronic neutropenia, exocrine pancreatic insufficiency (abnormal fecal elastase/fecal fat), metaphyseal skeletal dysplasia; iso(7q) and del(20q) common	MDS/AML ∼30%–35% lifetime; monosomy 7 high-risk, iso(7q) often indolent
*GATA2* deficiency	*GATA2*; AD (often *de novo*)	Monocytopenia, B-/NK-lymphopenia; recurrent NTM, fungal and HPV-associated infections; lymphedema; pulmonary alveolar proteinosis; often presents in adolescence/adulthood	MDS/AML ∼70% by age 70
*SAMD9*/*SAMD9L*-related disorders	*SAMD9*, *SAMD9L* (gain-of-function); AD	MIRAGE syndrome (*SAMD9*) or ataxia-pancytopenia syndrome (*SAMD9L*); transient or persistent monosomy 7; frequent somatic genetic rescue	Progression to MDS-EB in a subset; spontaneous remission possible in young children
Congenital amegakaryocytic thrombocytopenia	*MPL*; AR	Isolated severe thrombocytopenia at birth, absent/reduced megakaryocytes, elevated TPO; no congenital anomalies; progresses to pancytopenia	Progressive aplasia is the dominant risk; MDS/AML reported but less prominent than progression to BMF
Severe congenital neutropenia	*ELANE* (∼50%–60%, AD), *HAX1*, *G6PC3*, *JAGN1* (AR), *GFI1*, *CSF3R*	Severe neutropenia (ANC <0.2 × 10^9^/L) from early infancy with promyelocyte maturation arrest; recurrent bacterial infections; responsive to G-CSF	MDS/AML 10%–30% over 15y on G-CSF, associated with acquired *CSF3R*/*RUNX1* mutations

AD, autosomal dominant; AR, autosomal recessive; XLR, X-linked recessive; BMF, bone marrow failure; PF, pulmonary fibrosis; eADA, erythrocyte adenosine deaminase; TPO, thrombopoietin.

Clinically, FA manifests as progressive pancytopenia typically developing between ages 5 and 10 years, preceded by macrocytosis and elevated fetal hemoglobin. Congenital anomalies are present in approximately 75% of patients and include radial ray defects (absent or hypoplastic thumbs and radii), renal malformations, short stature, café-au-lait macules, and microcephaly. Approximately 25%–30% of patients lack any congenital anomaly and are diagnosed only upon investigation of cytopenia, underscoring the value of DEB/MMC testing in children with unexplained marrow failure.

The malignant risk in FA is exceptionally high: cumulative risk of MDS/AML exceeds 30% by age 40, and the lifetime risk of solid tumors—particularly squamous cell carcinomas of the head and neck and anogenital region—approaches 30%–50% in those who survive hematologic complications (10, 11). Extreme sensitivity to DNA cross-linking agents and ionizing radiation is a defining and clinically important feature that requires careful modification of diagnostic and therapeutic exposures. As in telomere biology disorders, androgen therapy (e.g., oxymetholone or danazol) can produce a temporary but clinically meaningful improvement in cytopenias in FA and is a useful bridge for patients without an available HSCT donor, although long-term use is limited by virilization, hepatotoxicity, and hepatic adenoma risk.

### Dyskeratosis congenita and telomere biology disorders

Telomere biology disorders (TBD) represent a clinical spectrum unified by pathologically short telomeres and encompass dyskeratosis congenita (DC), Hoyeraal–Hreidarsson syndrome, Revesz syndrome, and Coats plus syndrome, among others (25, 26). They are caused by mutations in at least 16 genes encoding telomerase components or telomere-protective proteins and may follow X-linked (DKC1), autosomal recessive (e.g., CTC1, PARN, NOP10), or autosomal dominant (TERC, TERT, TINF2, RTEL1, ACD, POT1) inheritance. Several of these genes do not map cleanly onto a single inheritance pattern: TERT, RTEL1, PARN, and ACD/TPP1 can each cause both autosomal recessive and autosomal dominant disease depending on zygosity and variant type (27).

The classic clinical triad of DC consists of reticular skin hyperpigmentation, nail dystrophy, and oral leukoplakia, but this triad is present in fewer than half of patients, and clinical recognition is further complicated by marked intrafamilial phenotypic variability. Hematologic manifestations range from isolated cytopenia to severe aplastic anemia and constitute the dominant cause of early mortality in childhood. Multisystem involvement—particularly pulmonary fibrosis, liver cirrhosis, and intestinal disease—is increasingly recognized in adults with TBD and significantly affects long-term prognosis, although these complications are uncommon as presenting features in children (25). Conditioning intensity in TBD requires individualized assessment: myeloablative regimens carry substantial risk of pulmonary and hepatic toxicity in patients with short telomeres, and fludarabine-based reduced-intensity protocols are generally favored, but the optimal approach depends on patient age, telomere length, pre-existing pulmonary or hepatic involvement, disease status, and the specific conditioning platform used, and should be determined on a case-by-case basis in consultation with an experienced transplant center (16). As in Fanconi anemia, androgens may provide temporary hematologic stabilization.

### Diamond-blackfan anemia

Diamond-Blackfan anemia (DBA) is a congenital erythroid aplasia caused by haploinsufficiency of ribosomal protein genes, most commonly RPS19 (approximately 25% of cases), RPL5, and RPL11, with over 20 causative genes identified to date (28); a 472-patient whole-exome sequencing cohort identified a likely causal mutation, most often in a ribosomal protein gene, in 78% of individuals (38). The resulting impairment of ribosome biogenesis activates p53-dependent pro-apoptotic pathways selectively in erythroid progenitors, producing pure red cell aplasia while initially sparing granulopoiesis and megakaryopoiesis.

DBA typically presents within the first year of life with severe macrocytic anemia, reticulocytopenia, and elevated erythrocyte adenosine deaminase (eADA) activity, the last being a diagnostically useful biomarker. Congenital anomalies are present in approximately 50% of patients and include craniofacial abnormalities (cleft palate, hypertelorism), upper limb defects, cardiac malformations, and urogenital anomalies. Thumb abnormalities in DBA are typically triphalangeal rather than absent, in contrast to FA.

First-line treatment consists of corticosteroids, which induce remission in approximately 80% of patients, though long-term use is limited by growth retardation, osteoporosis, and metabolic complications. Steroid-refractory or steroid-dependent patients require chronic transfusion support or HSCT. Eltrombopag has been used empirically as a steroid-sparing agent in small case series, but evidence supporting its efficacy in DBA remains limited and it is not established as standard therapy; luspatercept is under separate investigation for erythroid stimulation. Annual MDS/AML surveillance is recommended from adolescence.

### Shwachman-diamond syndrome

Shwachman-Diamond syndrome (SDS) is a multisystem disorder caused predominantly by biallelic mutations in SBDS (90% of cases), with DNAJC21, EFL1, and SRP54 accounting for minority cases (29). SBDS cooperates with EFL1 in the GTPase-driven release of eIF6 from the 60S ribosomal subunit, a critical step in ribosome maturation and translational activation. Impaired ribosome function leads to apoptosis of hematopoietic progenitors and acinar pancreatic cells.

Clinically, SDS is characterized by the triad of chronic neutropenia, exocrine pancreatic insufficiency (manifesting as steatorrhea and malabsorption in infancy), and skeletal dysplasia (metaphyseal chondrodysplasia on imaging). Hematologic abnormalities may affect all lineages and progress over time. Bone marrow cytogenetics characteristically reveal recurrent structural abnormalities, most notably isochromosome 7q [iso(7q)] and del(20q). Iso(7q), once thought uniformly alarming, may represent a clonal adaptation associated with favorable p53 pathway dynamics in some patients, but requires close longitudinal monitoring; monosomy 7 or complex cytogenetic changes indicate high malignant risk. Longitudinal sequencing of hematopoietic clones in SDS has shown that somatic EIF6 mutations—or the recurrent del(20q), which removes one copy of EIF6 and achieves the same effect at the chromosomal level—partially compensate for the underlying ribosome defect and mark a comparatively indolent, compensatory clonal path, whereas acquisition of biallelic TP53 alterations marks a maladaptive path with substantially higher leukemic potential, providing a mechanistic basis for prioritizing TP53 status in clonal surveillance (39).

HSCT corrects the hematologic component but does not address pancreatic insufficiency or skeletal disease. Conditioning must be individualized to account for multi-organ dysfunction: reduced-intensity regimens are generally preferred to minimize hepatic and cardiac toxicity, particularly in patients with DNAJC21 mutations, which are associated with early-onset cardiac complications.

### GATA2 deficiency

GATA2 deficiency arises from heterozygous loss-of-function mutations in the GATA2 gene, encoding a transcription factor critical for hematopoietic stem and progenitor cell differentiation (9, 30). The syndrome is now recognized to encompass previously described clinical entities including MonoMAC syndrome, Emberger syndrome (lymphedema with MDS), and familial MDS/AML, which represent phenotypic manifestations of the same molecular lesion.

A hallmark of GATA2 deficiency is a distinctive pattern of selective cytopenias: monocytopenia is nearly universal and often dramatic, frequently accompanied by depletion of B lymphocytes and NK cells while neutrophil and T cell counts may be relatively preserved. This immunological signature predisposes to a characteristic spectrum of infections including disseminated NTM, invasive fungal disease, and HPV-associated anogenital disease (30, 40). The magnitude of HPV-related solid tumor risk in GATA2 deficiency is not well defined and is likely to evolve further with increasing population HPV vaccination coverage; nonetheless, gynecologic/anogenital surveillance and HPV vaccination are recommended. Detection of peripheral monocytopenia below 0.1 × 10^9^/L, confirmed by flow cytometry with concurrent depletion of NK and B cells, is highly specific and should prompt GATA2 sequencing.

The risk of MDS and AML transformation reaches approximately 70% by age 70, and progression may occur even in patients with relatively mild cytopenias. Pre-emptive HSCT is increasingly recommended in patients with confirmed GATA2 deficiency, even prior to development of MDS, particularly in the presence of monosomy 7 or other high-risk cytogenetic abnormalities. Standard myeloablative conditioning is preferred when feasible, given the very high malignant risk. HSCT reverses most of the non-hematologic phenotype as well: it restores monocyte, B-cell, and NK-cell numbers, resolves recurrent NTM and fungal infections, and—through reconstitution of the alveolar macrophage compartment—has been reported to markedly improve pulmonary function in patients with pulmonary alveolar proteinosis (40). However, HSCT does not correct lymphedema and is not uniformly effective against established HPV-associated disease, underscoring the need for ongoing dermatologic and gynecologic surveillance.

### SAMD9/SAMD9L-related disorders

Germline gain-of-function mutations in SAMD9 or SAMD9L, two adjacent genes on chromosome 7q21.2, cause a spectrum of disorders that overlaps substantially with GATA2 deficiency and with pediatric MDS bearing monosomy 7 (31). SAMD9 mutations cause MIRAGE syndrome (myelodysplasia, infection, restriction of growth, adrenal hypoplasia, genital phenotypes, enteropathy), a severe multisystem disorder with high early mortality, while SAMD9L mutations cause ataxia-pancytopenia syndrome, characterized by progressive cerebellar ataxia with variable cytopenia, and a substantial proportion of apparently isolated pediatric MDS/aplastic anemia with monosomy 7 (31). Because affected hematopoietic stem cells are subject to strong selective pressure, somatic “rescue” events—loss of the mutant chromosome 7 (monosomy 7), acquisition of uniparental disomy 7q restoring two wild-type alleles, or a second somatic mutation in cis that inactivates the mutant allele—are extremely common and can lead to spontaneous, sometimes durable, hematologic remission in young children (31, 41). This phenomenon means that monosomy 7 in a young child with SAMD9/SAMD9L syndrome does not carry the same uniformly poor prognosis as monosomy 7 in other contexts, and a period of close observation rather than immediate HSCT may be appropriate in selected patients, although some clones progress to MDS with excess blasts and require timely transplantation (41). Because somatic reversion in blood and marrow can mask the germline variant, genetic testing should be performed on a non-hematopoietic tissue such as cultured skin fibroblasts. Given the overlap with GATA2 deficiency, SAMD9/SAMD9L should be included in any genetic testing panel ordered for suspected GATA2-related disease, particularly in children with monosomy 7 who lack the immunological phenotype of GATA2 deficiency.

### Congenital amegakaryocytic thrombocytopenia

Congenital amegakaryocytic thrombocytopenia (CAMT) is an autosomal recessive IBMFS caused by biallelic loss-of-function mutations in MPL, the gene encoding the thrombopoietin receptor (42). Of all IBMFS, CAMT is the most likely to present in the neonatal period: affected infants are typically born without congenital anomalies but have isolated, often severe, thrombocytopenia with absent or markedly reduced bone marrow megakaryocytes, frequently accompanied by intracranial or other serious bleeding. Serum thrombopoietin levels are markedly elevated, reflecting receptor unresponsiveness. Although thrombocytopenia is the presenting and initially isolated finding, the natural history is typically one of progressive trilineage marrow failure: most patients develop neutropenia and anemia within the first years of life and progress to severe aplastic anemia, with the rate of progression correlating with the residual functional activity of the mutant receptor (42). Management is largely supportive, with platelet transfusions and antifibrinolytics for bleeding episodes; thrombopoietin receptor agonists have not been shown to be effective, consistent with the underlying receptor defect. HSCT is the only curative therapy; given the general trajectory toward progressive marrow failure, transplantation is generally recommended once a suitable donor is identified, although timing may be individualized in patients with apparently milder mutations and a slower rate of progression (42).

### Severe congenital neutropenia

Severe congenital neutropenia (SCN) is a heterogeneous group of disorders characterized by a maturation arrest of myeloid precursors at the promyelocyte/myelocyte stage, resulting in profound peripheral neutropenia (absolute neutrophil count typically <0.2 × 10^9^/L) and recurrent, often life-threatening, bacterial infections from early infancy (43). Autosomal dominant mutations in ELANE, encoding neutrophil elastase, account for approximately 50%–60% of cases; autosomal recessive mutations in HAX1 (historically termed Kostmann syndrome), G6PC3, and JAGN1, and mutations in GFI1, WAS, and CSF3R, account for most of the remainder, though a substantial minority of patients remain genetically unsolved despite comprehensive sequencing (43). Daily granulocyte colony-stimulating factor (G-CSF) restores neutrophil counts and dramatically reduces infection-related mortality in most patients, but long-term G-CSF use is associated with a cumulative risk of progression to MDS/AML on the order of 10%–30% over 15 years, related at least in part to acquisition of somatic CSF3R and subsequently RUNX1 mutations (43). Patients with SCN therefore require regular monitoring of marrow morphology and cytogenetics, with HSCT reserved for those with G-CSF-refractory neutropenia (requiring very high doses without adequate response), clonal evolution to MDS/AML, or acquisition of high-risk somatic mutations.

## Treatment and hematopoietic stem cell transplantation

### General principles of management

Management of IBMFS is syndrome-specific and individualized, encompassing supportive care, pharmacological interventions, long-term surveillance, and curative HSCT. Therapeutic goals extend beyond correction of cytopenias to include prevention and early detection of clonal evolution, management of extrahematologic complications, and optimization of quality of life across decades of follow-up. Supportive therapy includes transfusion support, infection prophylaxis, and growth factor supplementation; G-CSF should be used cautiously, with attention to dose and clonal monitoring, in SDS, DC, and SCN given concerns about promoting clonal expansion.

### HSCT: indications, timing, and conditioning

HSCT is the only curative option for the hematologic component of the major IBMFS. Indications include severe or progressive marrow failure, transfusion dependence, life-threatening neutropenia with recurrent serious infections, and development of MDS or AML (15–17). As discussed above, pre-emptive HSCT before frank MDS evolution is increasingly supported in GATA2 deficiency and high-risk SDS. In CAMT, HSCT is generally undertaken proactively once a suitable donor is identified rather than deferred until pancytopenia develops. In SAMD9/SAMD9L syndrome, the timing decision is reversed for many young children with monosomy 7, in whom a period of close hematologic and molecular surveillance for spontaneous somatic rescue may be preferable to immediate transplantation (41). In SCN, HSCT is reserved for G-CSF-refractory disease or clonal evolution rather than offered upfront.

Optimal timing of HSCT must balance the risk of clonal evolution and irreversible organ damage against transplant-related morbidity, which varies substantially across syndromes. The benefit of HSCT in preventing MDS/AML transformation is well established in FA; however, outcomes are generally best when transplantation is performed before development of MDS, reinforcing the importance of regular surveillance and proactive transplant planning.

Table 3 summarizes key transplantation considerations by syndrome.

**Table 3:** Syndrome-specific considerations for hematopoietic stem cell transplantation.

Syndrome	Conditioning considerations	Timing considerations	Related-donor screening
Fanconi anemia	Reduced-intensity, fludarabine-based; avoid standard-dose alkylators/irradiation due to DNA crosslink hypersensitivity	Best outcomes when performed before MDS/AML develops	DEB/MMC breakage testing of siblings to exclude FA
Telomere biology disorders/DC	Fludarabine-based RIC; standard myeloablative regimens contraindicated due to pulmonary/hepatic toxicity in patients with short telomeres	Before significant pulmonary/hepatic dysfunction develops (mainly an adult-survivor concern)	Telomere length/full TBD gene panel in related donors (AD genes: *TERC*, *TERT*, *TINF2*, *RTEL1*)
Diamond–Blackfan anemia	Standard RIC or MAC per institutional protocol; no specific drug hypersensitivity	Steroid-refractory/transfusion-dependent disease or MDS/AML evolution	Screen siblings for the family ribosomal protein gene variant (variable penetrance)
Shwachman–Diamond syndrome	RIC generally preferred to limit hepatic/cardiac toxicity, particularly with *DNAJC21* mutations (early cardiac complications)	Severe/progressive marrow failure or high-risk clonal evolution (monosomy 7, complex karyotype); HSCT does not correct pancreatic/skeletal disease	Standard sibling testing (autosomal recessive)
*GATA2* deficiency	Myeloablative preferred when feasible, given high malignant risk	Pre-emptive, before overt MDS, especially with monosomy 7; also restores immune/pulmonary phenotype	*GATA2* (and *SAMD9*/*SAMD9L*) testing of relatives (autosomal dominant, variable penetrance)
*SAMD9*/*SAMD9L*-related disorders	Individualized; conditioning intensity guided by disease stage at transplant	In young children with monosomy 7, surveillance for spontaneous somatic rescue may be preferred over immediate HSCT; transplant for MDS-EB or progressive disease	*SAMD9*/*SAMD9L* testing of relatives on non-hematopoietic tissue (somatic reversion can mask variant in blood)
Congenital amegakaryocytic thrombocytopenia	Standard myeloablative regimens generally well tolerated (no DNA-repair or telomere defect)	Proceed once a donor is identified; do not wait for pancytopenia to develop	*MPL* carrier testing of siblings (autosomal recessive, carriers asymptomatic)
Severe congenital neutropenia	Standard conditioning per protocol; no specific hypersensitivity described	Reserved for G-CSF-refractory neutropenia or clonal evolution (monosomy 7, *CSF3R*/*RUNX1* mutations); not first-line	*ELANE* (AD) or *HAX1*/*G6PC3* (AR) testing of relatives as appropriate

RIC, reduced-intensity conditioning; MAC, myeloablative conditioning; MDS-EB, myelodysplastic syndrome with excess blasts.

Selection of an HSCT donor requires genetic evaluation of related donors to exclude asymptomatic carriers, particularly important in autosomal dominant disorders (TERC, TERT, TINF2, GATA2), in dominantly inherited SAMD9/SAMD9L syndrome, and in FA (where siblings should undergo DEB/MMC testing). As discussed in the Introduction, the use of an unrecognized mutation-carrying relative as a donor is a serious and avoidable pitfall, and germline testing of potential related donors—performed on a non-hematopoietic tissue where somatic reversion is a concern, as in SAMD9/SAMD9L syndrome—should precede donor confirmation whenever feasible. Outcomes with matched unrelated donors are now comparable to sibling donor outcomes in most IBMFS. Haploidentical transplantation with post-transplant cyclophosphamide is increasingly used when a matched donor is unavailable.

### Post-transplant surveillance

HSCT corrects the hematologic phenotype but does not eliminate the underlying germline defect from non-hematopoietic tissues. Long-term surveillance for non-hematologic malignancies, organ-specific complications, and immune reconstitution should therefore continue over the long term in HSCT recipients with an IBMFS diagnosis. Annual screening for squamous cell carcinomas of the head and neck and anogenital region is strongly recommended in FA survivors (10, 11). Pulmonary, hepatic, and skin surveillance remain important in DC/TBD, particularly for adult survivors. Lifelong HPV screening and vaccination optimization are indicated in GATA2 deficiency, recognizing that the magnitude of residual HPV-related risk after HSCT and vaccination remains an area of active study.

## Future directions

Gene therapy has advanced furthest in Fanconi anemia. Autologous, lentiviral vector-mediated correction of FANCA in CD34+ hematopoietic stem cells (RP-L102, building on the academic FANCOLEN-1 program) does not require cytotoxic conditioning, since corrected cells acquire an intrinsic proliferative advantage over the FA-deficient background; open-label phase 1/2 and long-term follow-up data have demonstrated durable vector-marked engraftment and reversal of bone marrow failure in a majority of treated patients, with no genotoxic events reported to date (44). Gene editing in FA, however, carries a distinctive and almost circular obstacle: conventional CRISPR-Cas9 correction by homology-directed repair relies on the cell's own DNA interstrand crosslink repair machinery to complete the edit at the Cas9-induced double-strand break, and that machinery is the FA pathway itself—meaning the very defect gene therapy aims to correct is also required to install the correction efficiently (45). Investigators have begun to work around this circularity by exploiting non-homologous end joining, which is relatively enhanced in FA cells and in primitive hematopoietic stem cells generally, to generate small compensatory insertions/deletions that restore the reading frame of the mutant gene without requiring the FA pathway (46); base and prime editing, which do not create double-strand breaks, are also under early investigation as potential routes around this constraint. Gene therapy approaches for Diamond–Blackfan anemia, targeting the causative ribosomal protein haploinsufficiency, remain earlier in preclinical development.

Targeted pharmacological interventions are under active investigation across several syndromes: eltrombopag has produced hematologic responses in small series of steroid-refractory DBA, although robust evidence is still lacking; luspatercept is under investigation for erythroid stimulation in DBA; and danazol provides temporary hematologic stabilization in both telomere biology disorders and Fanconi anemia, with telomerase-directed pharmacological approaches remaining largely investigational. Error-corrected deep sequencing and machine learning-based clonal evolution models are improving risk stratification for malignant transformation, while international patient registries and broader adoption of genome sequencing continue to expand diagnostic yield and may help resolve some of the panel-negative and variant-of-uncertain-significance cases discussed above. For SAMD9/SAMD9L syndrome, refinement of criteria for “watch and wait” surveillance vs. pre-emptive HSCT in young children with transient monosomy 7 remains an active area of clinical research (31).

## Conclusion

Inherited bone marrow failure syndromes constitute a clinically and biologically heterogeneous group of childhood disorders in which distinct molecular defects—affecting DNA repair, telomere maintenance, ribosome biogenesis, or transcriptional regulation—converge on a shared pathway of hematopoietic stem cell exhaustion, progressive cytopenias, and markedly elevated risk of myeloid malignancy.

The central diagnostic challenge lies in their distinction from acquired marrow failure in patients lacking overt syndromic features, a challenge with direct and potentially serious therapeutic consequences. Systematic integration of clinical red flags, targeted functional assays, and germline NGS panels into the evaluation of children with unexplained persistent cytopenia is therefore important.

Contemporary management requires individualized, multidisciplinary care integrating supportive therapy, syndrome-specific pharmacological interventions, clonal surveillance, and timely HSCT with adapted conditioning. Recognition that HSCT corrects only the hematologic phenotype supports the need for long-term follow-up in affected patients.

Ongoing advances in molecular diagnostics, gene therapy, targeted pharmacology, and risk-adapted transplant strategies offer realistic prospects for improving long-term outcomes. Realization of this potential will require sustained investment in prospective international registries and collaborative clinical trials to generate the evidence base currently lacking for many of these rare conditions.
